# A Neuron-Specific Antiviral Mechanism Modulates the Persistent Infection of Rice Rhabdoviruses in Leafhopper Vectors

**DOI:** 10.3389/fmicb.2020.00513

**Published:** 2020-04-17

**Authors:** Haitao Wang, Ye Liu, Lining Mo, Chenyang Huo, Ziyao Wang, Panpan Zhong, Dongsheng Jia, Xiaofeng Zhang, Qian Chen, Hongyan Chen, Taiyun Wei

**Affiliations:** Fujian Province Key Laboratory of Plant Virology, Vector-Borne Virus Research Center, College of Plant Protection, Fujian Agriculture and Forestry University, Fuzhou, China

**Keywords:** antiviral mechanism, central nervous system, *Hikaru genki*, rice rhabdoviruses, rice leafhopper, rice yellow stunt virus, matrix protein

## Abstract

Many plant rhabdoviruses are neurotropic and can persistently infect the central nervous system (CNS) of their insect vectors without causing significant cytopathology. The mechanisms by which the insect CNS resists infection by plant rhabdoviruses are largely unknown. Here, we report that the neural factor *Hikaru genki* homolog of the leafhopper *Nephotettix cincticeps* (NcHig) limits the spread of the nucleorhabdovirus rice yellow stunt virus (RYSV) in vector CNS. NcHig is predominantly expressed in the CNS of *N. cincticeps*, and the knockdown of NcHig expression by RNA interference enhances RYSV infection of the CNS. Furthermore, immuno-blockade of NcHig function by microinjection of *N. cincticeps* with NcHig antibody also enhances viral infection of the CNS. Thus, we conclude that the neuron-specific factor NcHig can control RYSV propagation in the CNS. Interestingly, we find the Hig homolog of the leafhopper *Recilia dorsalis* also has antiviral activity during the persistent infection of the cytorhabdovirus rice stripe mosaic virus (RSMV) in vector CNS. We further determine that RYSV and RSMV matrix proteins specifically interact with the complement control protein (CCP) domains of Higs. Thus, the matrix protein-binding ability of Hig is potentially essential for its antiviral activity in rice leafhoppers. Our results demonstrate an evolutionarily conserved antiviral mechanism for Hig to mediate the persistent infection of rice rhabdoviruses in the CNS of leafhopper vectors.

## Introduction

Many plant viruses that cause substantial agricultural losses, such as reoviruses, tospoviruses, tenuiviruses, and rhabdoviruses, are transmitted by insect vectors in a persistent-propagative manner (Hogenhout et al., 2008; Ammar et al., 2009; Wei and Li, 2016). Persistent viruses, after being acquired from plant phloem, initially infect the insect intestines and then spread to the hemolymph. From the hemolymph, persistent viruses eventually invade the salivary glands to be horizontally transmitted to healthy plants, or only few are vertically transmitted to the offspring (Hogenhout et al., 2008; Ammar et al., 2009; Wei and Li, 2016). Several plant rhabdoviruses, such as maize mosaic virus, maize fine stripe virus, rice yellow stunt virus (RYSV), and rice stripe mosaic virus (RSMV), are neurotropic and persistently infect the nervous system of their insect vectors (Ammar and Hogenhout, 2008; Todd et al., 2010; Wang et al., 2019; Zhao et al., 2019). Although many plant rhabdoviruses can infect, propagate in, and spread through the nervous system of insect vectors, the molecular mechanisms involved in the persistent viral propagation within the neurons remain poorly understood.

Rhabdoviruses form a large family whose collective host ranges include vertebrates, invertebrates, and plants, and are of considerable socioeconomic and agricultural importance (Dietzgen and Kuzmin, 2012). For example, leafhopper-transmitted RYSV, a nucleorhabdovirus, and RSMV, a cytorhabdovirus, pose serious agricultural threats in Asian rice-growing countries (Chiu et al., 1965; Fan et al., 1965; Yang et al., 2017a, b; Chen S. et al., 2019). Typical rhabdoviruses are single-stranded RNA viruses with non-segmented negative-sense genomes encoding five structural proteins, which form bullet-shaped or bacilliform virions (Walker et al., 2018). During viral assembly, nucleoprotein (N), polymerase (L), phosphoprotein (P), and the RNA genome form a ribonucleoprotein (RNP) core (Dietzgen et al., 2017). The rhabdovirus matrix (M) protein, a small (20–25 kDa) self-association protein, condenses RNP cores to form non-enveloped viral particles (Jayakar et al., 2004; Kuzmin et al., 2009). It has been proposed that the replication and assembly of rhabdoviruses occur in viral inclusions called viroplasms in the nucleus or cytoplasm of infected cells (Walker et al., 2018). Generally, RNP cores are constructed inside the viroplasms, whereas M proteins are added to the RNP cores at the periphery of the viroplasms to produce non-enveloped virions (Ammar et al., 2009). The enveloped virions are assembled at the endoplasmic reticulum (ER) or the inner nuclear membranes in plant cells, or at the plasma membrane in animal cells (Gaedigk et al., 1986; Jayakar et al., 2004; Jackson et al., 2005). How these viral proteins are involved in the persistent viral propagation of the nervous system in insect vectors is still poorly understood.

The neurotropic nature of plant rhabdoviruses suggests that there might be an alternative non-hemolymph pathway that allows the rapid spread of viruses from the initial infection site in the intestinal epithelium to the salivary glands via nerve networks (Ammar and Hogenhout, 2008; Whitfield et al., 2018; Wang et al., 2019; Zhao et al., 2019). We recently observed that RYSV M protein interacted with axonal microtubules to mediate the rapid movement of the virions along axons in the insect central nervous system (CNS), thereby facilitating the persistent virus transmission by leafhopper vectors (Wang et al., 2019). Several plant rhabdoviruses can multiply to a high viral titer in the CNS of their vectors, in which viral infection is apparent and persistent (Chiu, 1982; Ammar and Hogenhout, 2008; Whitfield et al., 2018; Wang et al., 2019; Zhao et al., 2019). The persistent infection of plant rhabdoviruses generally does not cause cytopathologic changes in insect CNS; however, the neuro-invasive viruses of vertebrates generally cause degeneration of axons, cellular pathogenesis, and even neurologic disease or similar neuroinflammatory disorders (McArthur et al., 2005; Spudich and González-Scarano, 2012; Avdoshina et al., 2017; Leibovitch and Jacobson, 2018). Thus, it is important to understand how plant rhabdoviruses modulate their persistent infection in the CNS of insect vectors.

In this study, we used rice rhabdoviruses (RYSV and RSMV) and their main leafhopper vectors, *Nephotettix cincticeps* and *Recilia dorsalis* (Hemiptera, Delphacidae), to explore how insect factors regulate the persistent infection of rhabdoviruses in the CNS of insect vectors. We report that the neuron-specific factors, the *Hikaru genki* (Hig) homologs of rice leafhoppers, directly interact with M proteins of rice rhabdoviruses and play a conserved antiviral role in controlling viral infection in the CNS of leafhopper vectors.

## Materials and Methods

### Insects, Viruses, and Antibody

Rice leafhoppers (*N. cincticeps* and *R. dorsalis*) were collected from Yunnan Province in southern China and reared on rice seedlings in cages in a controlled environment at 28°C with 75 ± 5% humidity and a 16 h light/8 h dark cycle. The rice leafhoppers were reared on rice seedlings in a controlled environment as described previously (Wang et al., 2019). RYSV-infected rice samples were propagated via transmission by RYSV-infected *N. cincticeps* (Wang et al., 2019). RSMV-infected rice plants were collected from rice fields in Luoding, Guangdong Province, China, and maintained on rice plants via transmission by *R. dorsalis*. RYSV and RSMV were crudely purified from infected rice plants, as described by Wei et al. (2006). Rabbit polyclonal antibody against N and M proteins encoded by RYSV and RSMV were prepared as previously described (Wang et al., 2018; Zhao et al., 2019). The α-tubulin antibody (Sigma), a general marker of neural structures, was used to label the leafhopper CNS. Polyclonal antibody was conjugated directly to fluorescein isothiocyanate (FITC) or rhodamine according to the manufacturer’s instructions.

### Bioinformatics

The sequences of the Hig genes from *N. cincticeps* (NcHig) or *R. dorsalis* (RdHig) were obtained from our transcriptome. The functional modules of Hig genes from *N. cincticeps* (GenBank accession no.MN815919), *Aedes aegypti* (AaHig) (GenBank accession no. AIS74715), and *Drosophila melanogaster* (DmHig) (GenBank accession no. NP_724772.1) were predicted using SMART^1^ and Pfam^2^. The sequences of the Hig genes of agricultural pest insects were obtained from the NCBI database. The unrooted phylogenetic tree was built with the neighbor-joining method using MEGA software based on the alignment of the sequences determined using CLUSTAL W. The boot-strap consensus tree was inferred from 5000 replicates.

### Yeast Two-Hybrid Assay

A yeast two-hybrid assay was performed using the Matchmaker Gal4 Two-Hybrid System 3 (Takara Bio), according to the manufacturer’s instructions. The RYSV M gene (GenBank accession no. NP_620499.1) was amplified and cloned into the bait vector pGBKT7 (pGBKT7-M), and different NcHig domains were cloned into the prey vector pGADT7 (pGADT7-Nc-CCP1, pGADT7-Nc-IG, pGADT7-Nc-CCP2, pGADT7-NcHig-full length). The recombinant vectors pGBKT7-M/pGADT7-Nc-CCP1, pGBKT7-M/pGADT7-Nc-IG, pGBKT7-M/pGADT7-Nc-CCP2, and pGBKT7-M/pGADT7-NcHig-full length as well as the positive control pGBKT7-53/pGADT7-T and the negative control pGBKT7-Lam/pGADT7-T were each co-transformed into the AH109 yeast strain, respectively. The segment containing the complement control protein (CCP) domain at the C-terminus (396-631 aa, CCP2) of *R. dorsalis* Hig (RdHig-CCP2) (GenBank accession no. MT043161) was cloned into the pGADT7 vector (pGADT7-RdHig-CCP2). The RSMV M gene (Gene ID: 41700835) was cloned into pGBKT7.

To explore the interaction region of RYSV M protein with NcHig, full-length of RYSV M gene were divided into N-terminal (1-131 aa) and C-terminal (132-262 aa) fragments and cloned into the bait vector pGBKT7, respectively. The recombinant vectors pGBKT7-M-N-terminal/pGADT7-NcHig-CCP2, pGBKT7-M-C-terminal/pGADT7-NcHig-CCP2, as well as the positive control and the negative control were each co-transformed into the AH109 yeast strain, respectively. The plasmids were also transformed into the yeast cells to verify the interaction of RSMV with RdHig. β-galactosidase activity was assessed on SD/-Leu/-Trp/-His/-Ade/X-α-gal agar culture medium plates.

### GST Pull-Down Assay

A GST pull-down assay was performed to detect the interaction of RYSV M protein with NcHig. The RYSV M gene was amplified and cloned into the pGEX-3X vector, which included a GST-tag (M-GST). The NcHig CCP2 domains were cloned and inserted into a His-fusion vector, pDEST17 (His-NcHig-CCP2). The constructed plasmids pDEST17-NcHig-CCP2, pGEX-3X-M, and pGEX-3X (GST) were separately expressed in the *Escherichia coli* BL21 DE3 strain. The lysates of cells containing the pGEX-3X-M (M-GST) and pGEX-3X (GST) vectors were incubated with Glutathione Sepharose beads (Amersham) for 4 h at 4°C. The beads were rinsed with 0.01 M PBS to remove the redundant proteins, and were then incubated with lysates of cells containing pDEST17 (His-NcHig-CCP2) for a further 4 h at 4°C. The mixtures were then washed with elution buffer and visualized using an immunoblot assay with GST-tag and His-tag antibody (Abcam), respectively. The interaction of RSMV and RdHig was also detected by a GST pull-down assay.

### Expression Analysis of Hig in Rice Leafhoppers

To analyze the expression levels of NcHig in different tissues of *N. cincticeps*, the head, alimentary canal, ovary, and testis of adult instar leafhoppers were dissected for extraction of the total RNAs and proteins of different tissues. The relative expression of Hig (primers in Supplementary Table S1) in different tissues of adult *N. cincticeps* or *R. dorsalis* was detected with an RT-qPCR assay by using a QuantStudio^TM^ 5 Real-Time PCR machine. The detected transcript levels were normalized to the transcript levels of the internal control genes, i.e., actin or elongation factor 1 (EF1) alpha, and estimated using the 2^–Δ^ ^Δ^ ^Ct^ (cycle threshold) method.

To verify the expression patterns of Hig in different tissues of rice leafhoppers, the segment containing NcHig CCP2 domain was cloned into the pDONR221 vector and then recombined into the pDEST17 expression vector. The recombinant plasmid was expressed in the *E. coli* strain BL21. The lysates of cells containing the recombinant NcHig were purified using His-tag purification resin. The polyclonal antibody was produced by immunization of NcHig in mice prepared by the Beijing Protein Innovation Company, which was approved by the Beijing Municipal Science and Technology Commission. The specificity of antibody was detected by an immunoblot assay. Total proteins were extracted from leafhopper tissues and then analyzed by immunoblot assay.

### Effects of the Knockdown of Hig Expression by RNA Interference (RNAi) Strategy on Viral Infection in Rice Leafhoppers

Double-stranded RNAs (dsRNAs) of Hig (dsHig) and GFP (dsGFP) (primers in Supplementary Table S1) were synthesized *in vitro* as previously described (Wang et al., 2018). The mixtures of synthesized dsRNAs and virus solutions were microinjected into the third instar rice leafhoppers. The microinjected third instar insects were reared on healthy rice seedlings in containers under controlled conditions. Based on RYSV infection process within the body of *N. cincticeps* (Wang et al., 2019), at 3, 6, or 8 days post-microinjection, the alimentary canals and heads of rice leafhoppers were dissected for immunofluorescence microscopy, and the total RNAs were isolated to assess the transcript levels of Hig or viral genes using RT-qPCR assay. Three biological repeats were used for the RT-qPCR assay and analyzed using a Student’s *t*-test. The RYSV or RSMV genome copies (viral titers) in the individual head or alimentary canal of viruliferous leafhopper were calculated as the log of the copies number/μg head or alimentary canal RNAs based on the standard curve for the N genes of RYSV or RSMV (primer in Supplementary Table S1). Additionally, Hig and viral accumulations were analyzed by an immunoblot assay using Hig-, N-, or M-specific antibody, respectively.

For the survival assay, we microinjected the mixtures of synthesized dsRNAs and virus solutions into the third instar rice leafhoppers (*n* = 70) and monitored their survival daily.

### Immuno-Blockade Assay of Hig Function in Rice Leafhoppers

Immuno-blockade of the interaction of viruses with insect factors in various tissues such as brain, alimentary canal, and ovary by microinjection of specific antibody against insect factors has been extensively used (Xiao et al., 2015; Wu et al., 2019). In the immuno-blockade assay, the third instar rice leafhoppers were microinjected with the mixtures of NcHig antibody and virus solutions. The mixtures of pre-immune antibody and virus solutions were used as the control treatment. The microinjected third instar insects were then reared on healthy rice seedlings. At 3 or 6 days post-microinjection, rice leafhoppers were dissected for immunofluorescence microscopy. Total RNAs were isolated from the head or alimentary canal to quantify viral titers using RT-qPCR assay.

### Immunofluorescence Microscopy

Immunofluorescence microscopy was used to examine viral distribution in the CNSs of leafhoppers that had been microinjected with antibody or dsRNAs. The third instar rice leafhoppers were microinjected with antibody or dsRNAs and then transferred to healthy rice seedlings. At 6 days after microinjection, the alimentary canals and heads from 30 rice leafhoppers were dissected, fixed with 4% paraformaldehyde at room temperature for at least 8 h, and permeabilized in 4% Triton X-100 for 24 h. The treated heads were also immunolabeled with M antibody conjugated to rhodamine (M-rhodamine) and α-tubulin antibody conjugated to FITC (α-tubulin-FITC, Sigma). The alimentary canals were immunolabeled with M-rhodamine and the actin dye phalloidin-Alexa 488 (Invitrogen). The samples were then examined using a Leica TCS SP5II confocal microscope.

### Statistical Analysis

All data were analyzed using SPSS 19.0. The significance of the means between two samples was analyzed by a Student’s *t*-test.

## Results

### RYSV M Protein Interacts With a Homolog of Hig in *N. cincticeps*

**FIGURE 1:**
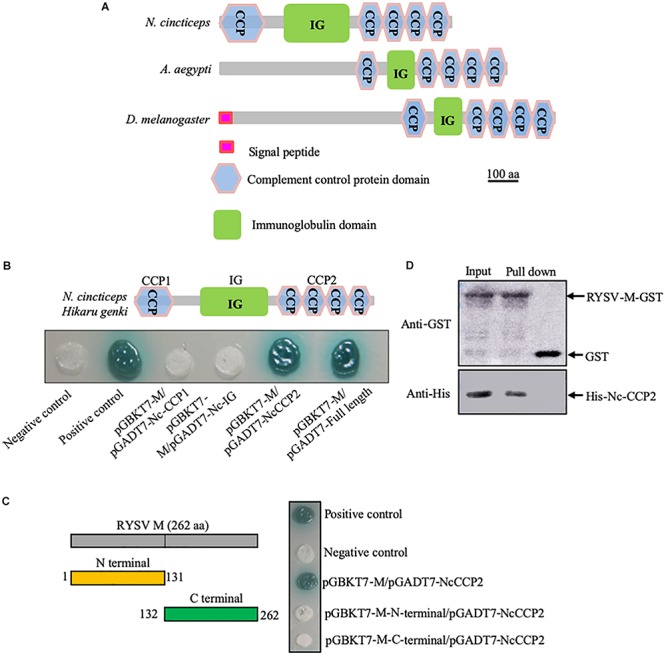
Interaction of RYSV M protein with a homolog of Hig in *N. cincticeps*. **(A)** Schematic representation of DmHig, AaHig, and NcHig protein domains. The proteins were predicted using the InterPro and Smart websites. The protein domains of NcHig divided into three parts, CCP1, IG, and CCP2. **(B)** Yeast two-hybrid assay to detect the interaction of RYSV M protein with the CCP2 domains of NcHig. **(C)** Yeast two-hybrid assay to detect the interaction of the NcHig CCP domains with N-terminal (1-131 aa) or C-terminal (132-262 aa) fragments of RYSV M protein. **(D)** GST pull-down assay to verify the interaction of the NcHig CCP2 domains with RYSV M protein. GST protein was used as the control.

To identify insect factors that interact with RYSV M, we screened a cDNA library of *N. cincticeps* using a yeast two-hybrid assay. This screen yielded 178 positive colonies, of which 62 were randomly sequenced. Of the resulting sequences, 27 candidate interactors were annotated using the BLASTX program in GenBank (Supplementary Table S2). These candidates included the neurons protein, the key regulators in vesicle formation or trafficking such as the transitional ER ATPase and Ras- or Rab-related proteins, and the tubulin (Supplementary Table S2). Furthermore, an *N. cincticeps* homolog of the neural factor Hig (NcHig) caught our attention. Hig is predominantly expressed in the pupal and adult nervous system of *Drosophila* and mosquitoes, and is involved in the resistance to pathogenic virus infection (Hoshino et al., 1996; Xiao et al., 2015). The full-length open reading frame (1911 bp) of NcHig was amplified and sequenced (GenBank accession no. MN815919). Sequence alignment indicated that NcHig shared 22.06% and 18.80% similarity with its counterparts AaHig and DmHig, respectively (Supplementary Figure S1A). Among agricultural insect pests with available genome sequences, Hig protein sequences are evolutionarily conserved, suggesting that these proteins also share similar functions (Supplementary Figure S1B). The predicted amino acid sequence (636 aa) of NcHig was analyzed and contained one putative immunoglobulin (IG) domain and five putative CCP domains (Figure 1A). The CCP domains are the signature features of many mammalian and insect complement proteins and can recognize pathogenic microorganisms (Xiao et al., 2014, 2015; Nakayama et al., 2016).

Yeast two-hybrid assay showed that RYSV M can specifically interact with the CCP domains at the C-terminus (396-631 aa, CCP2) of NcHig, but not with other domains (Figure 1B). However, both the N-terminal (1-131 aa) and C-terminal (132-262 aa) fragments of RYSV M did not interact with the CCP domains of NcHig (Figure 1C). A GST pull-down assay confirmed that RYSV M-GST could bind to CCP2-His, whereas GST could not (Figure 1D). Thus, NcHig CCP domains have the conserved role in recognizing RYSV M.

### NcHig Is Preferentially Expressed in the Central Nervous System (CNS) of *N. cincticeps*

**FIGURE 2:**
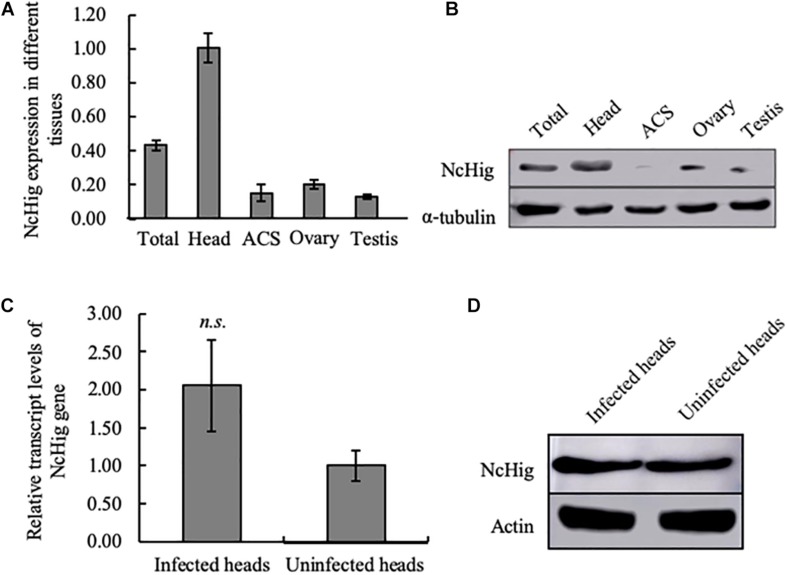
The specific expression of NcHig in the CNS of *N. cincticeps*. **(A)** RT-qPCR assay of NcHig expression in total insect (total) and the indicated tissues of *N. cincticeps*. The detected transcript levels were normalized to the transcript levels of internal control actin gene and estimated using the 2^–ΔΔ*Ct*^ (cycle threshold) method. ACS, alimentary canal. Values are the means (±SE) of three biological replicates. **(B)** Quantification of NcHig protein levels in the indicated tissues as determined by an immunoblot assay using NcHig-specific antibody. α-tubulin was used as the loading control. **(C)** Transcript levels of NcHig were normalized to the transcript levels of internal control actin gene in the virus-infected or uninfected heads of *N. cincticeps*, as detected by an RT-qPCR assay. Values are the means (±SE) of three biological replicates. **(D)** Accumulation of NcHig protein in the virus-infected or uninfected heads of *N. cincticeps*, as detected by an immunoblot assay. Insect β-actin was used as the internal control.

*Drosophila* and mosquito Hig proteins are specifically expressed in the CNS (Xiao et al., 2015). To establish whether this is also true of leafhopper Hig proteins, we assessed the abundance of NcHig transcript in different tissues of *N. cincticeps* using RT-qPCR assay. NcHig transcripts were highly abundant in the leafhopper head, where the CNS is located (Figure 2A). To further understand the distribution of NcHig protein, we expressed and purified a fragment including IG and CCP2 domains at the C-terminal of NcHig in *E. coli* and generated a polyclonal antibody in mice. This antibody could detect the Hig proteins from the heads of leafhoppers *N. cincticeps* or *R. dorsalis* (Supplementary Figure S2). Consistent with its mRNA expression pattern, the NcHig protein was predominantly expressed in the leafhopper heads, as detected by an immumoblot assay (Figure 2B). Thus, NcHig is preferentially expressed in the CNS of *N. cincticeps*.

We next examined whether the expression of NcHig in insect CNS was influenced by RYSV infection. RT-qPCR and immunoblot assays showed that viral infection caused no change in the abundance of NcHig mRNA or protein in leafhopper heads (Figures 2C,D), suggesting that RYSV infection of the leafhopper CNS did not activate Hig expression.

### Hig Impedes RYSV Infection of the CNS of *N. cincticeps*

**FIGURE 3:**
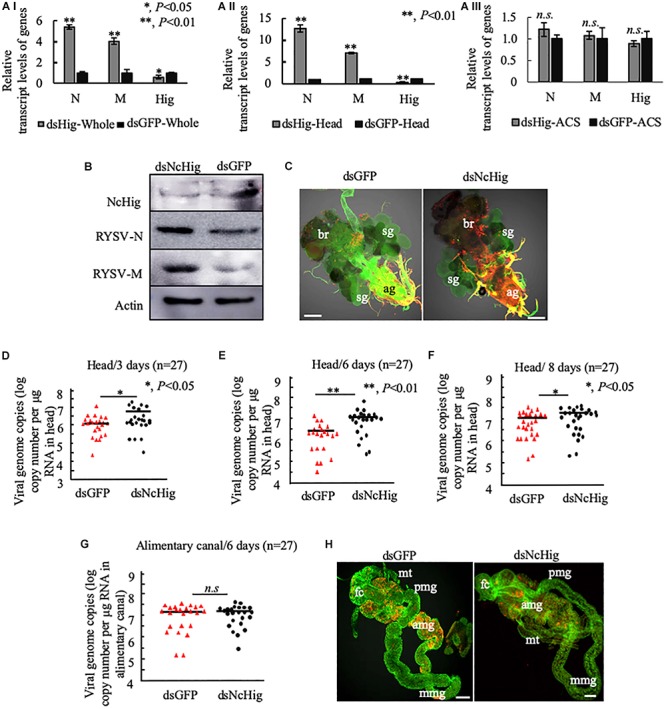
The dsNcHig-mediated knockdown of NcHig expression specifically impedes RYSV infection in the CNS of the *N. cincticeps* vector. **(A)** The transcript levels of RYSV N, RYSV M, and NcHig were normalized to the transcript levels of internal control actin gene in the whole body **(A-I)**, head **(A-II)**, and alimentary canal (ACS) **(A-III)** of viruliferous *N. cincticeps* at 6 days post-microinjection of dsNcHig and dsGFP, as detected by RT-qPCR assay. *P*-values were estimated using a Student’s *t*-test. **(B)** The accumulation levels of NcHig and RYSV N and M in the heads of viruliferous *N. cincticeps* at 6 days post-microinjection of dsNcHig and dsGFP, as detected by an immunoblot assay. Insect β-actin was used as an internal control. **(C)** RYSV infection of the CNS of viruliferous *N. cincticeps* at 6 days post dsRNAs microinjection was analyzed by immunofluorescence assay. Virus-infected heads were immunolabeled with RYSV-M-rhodamine (red) and α-tubulin-FITC (green). ag, abdominal ganglion; br, brain; sg, salivary gland. Bars, 50 μm. RT-qPCR assay was used to detect the viral genome copies of RYSV in the heads of *N. cincticeps* at 3 days **(D)**, 6 days **(E)**, or 8 days **(F)** post-microinjection of dsRNAs. The RYSV genome copies in the individual heads (*n* = 27) from viruliferous *N. cincticeps* were calculated as the log of the copies number/μg RNA in head based on the standard curve for the RYSV N gene. The results were analyzed from three biological repeats. *P*-values were estimated using a Student’s *t*-test. **(G)** RT-qPCR assay was used to detect the viral genome copies of RYSV in the alimentary canals of *N. cincticeps* at 6 days post-microinjection of dsRNAs. RYSV genome copies in the individual alimentary canal (*n* = 27) from viruliferous leafhoppers were calculated as the log of the copies number/μg RNA in alimentary canal based on the standard curve for the RYSV N gene. The results were analyzed from three biological repeats. **(H)** The infection of RYSV in the intestines of viruliferous leafhoppers at 6 days post dsRNAs microinjection was analyzed by immunofluorescence assay. Virus-infected alimentary canals were immunolabeled with RYSV-M-rhodamine and the actin dye phalloidin-Alexa Fluor 488 (green). fc, filter chamber; mt, midgut; amg, anterior midgut; mmg, middle midgut; pmg, posterior midgut. Bars, 50 μm.

We next investigated the functional role of NcHig during the persistent infection of RYSV in the CNS of *N. cincticeps* using RNA interference (RNAi) strategy. Specifically, we microinjected the leafhopper bodies with purified RYSV along with dsNcHig or dsGFP. At 6 days after microinjection, RT-qPCR and immunoblot assays showed that dsNcHig treatment significantly decreased NcHig expression in whole leafhopper bodies and heads at both the mRNA and protein levels (Figures 3A,B). Furthermore, knockdown of NcHig expression by the dsNcHig treatment significantly increased the transcript levels of RYSV N and M in the whole leafhopper bodies and heads, but not in the alimentary canal (Figure 3A). Immunoblot assay confirmed the significant increase in RYSV N and M protein levels in dsNcHig-treated heads (Figure 3B). Immunofluorescence microscopy showed that RYSV M was extensively distributed throughout the CNS in dsNcHig-treated leafhoppers, but was restricted to limited areas of the CNS in dsGFP-treated leafhoppers (Figure 3C). To quantitate the effect of NcHig on viral load, we measured the viral genome copies number in the heads of *N. cincticeps* at 3, 6, and 8 days after dsRNAs microinjection using RT-qPCR assay. The mean viral genome copy number was always higher in dsNcHig-treated groups than in dsGFP-treated groups (Figures 3D–F). Together, these observations indicate that NcHig plays an antiviral role during RYSV infection of the CNS of *N. cincticeps*.

We have shown that the knockdown of NcHig specifically enhanced viral infection in the head, but not in the alimentary canal of *N. cincticeps* (Figure 3A). Furthermore, the mean viral genome copies number in the alimentary canal of *N. cincticeps* was not significantly different between the dsNcHig- and dsGFP-treatments (Figure 3G). Immunofluorescence microscopy confirmed that RYSV infection of the leafhopper alimentary canals did not change significantly after dsNcHig treatment (Figure 3H). Thus, NcHig is a neuron-specific factor that controls viral propagation in the CNS but not in the peripheral intestinal tissues.

We then assessed whether the silencing of NcHig influences insect survival upon RYSV infection. The silencing of NcHig did not directly cause phenotypic abnormalities or death of non-viruliferous *N. cincticeps* (Supplementary Figure S3). However, after viral infection, a higher mortality rate was observed in dsNcHig-treated group than in dsGFP-treated group (Supplementary Figure S3). It seemed that an increase of viral accumulation in the CNS of *N. cincticeps* was associated with the increased mortality rate of dsNcHig-treated *N. cincticeps*. These results indicate that the NcHig effectively modulates the persistent infection of RYSV in the CNS of *N. cincticeps.*

### Immuno-Blockade of NcHig Promotes RYSV Propagation in the CNS of *N. cincticeps*

**FIGURE 4:**
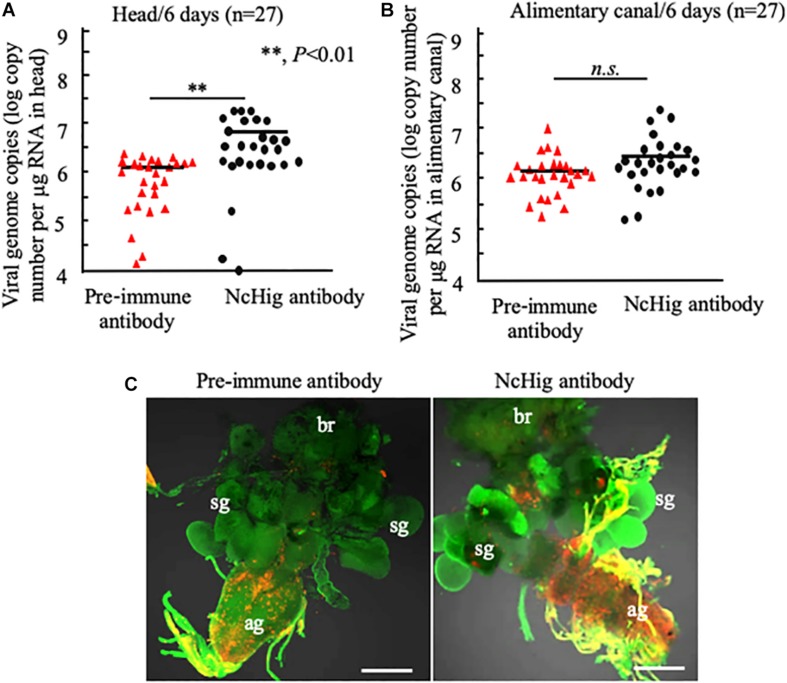
Immuno-blockade of NcHig enhanced the infection of RYSV in the CNS of *N. cincticeps*. RT-qPCR assay of viral genome copies of RYSV in the head **(A)** and alimentary canal **(B)** of *N. cincticeps* at 6 days post-microinjection of NcHig antibody or pre-immune antibody as the control. The RYSV genome copies in the individual samples (*n* = 27) from viruliferous *N. cincticeps* were calculated as the log of the copies number/μg tissue RNA based on the standard curve for the RYSV N gene. The analysis was based on three biological repeats. *P*-values were estimated using a Student’s *t*-test. **(C)** RYSV infection of the CNS of viruliferous *N. cincticeps* at 6 days post-microinjection of NcHig antibody or pre-immune antibody, as analyzed by an immunofluorescence assay. Virus-infected heads were immunolabeled with RYSV-M-rhodamine (red) and α-tubulin-FITC (green). ag, abdominal ganglion; br, brain; sg, salivary gland. Bars, 50 μm.

To further validate the antiviral role of NcHig during RYSV infection of vector CNS, we performed an immuno-blockade of NcHig function *in vivo* with NcHig antibody. We microinjected the NcHig antibody along with RYSV into the thorax of *N. cincticeps*, and subsequently measured RYSV genome copies number in the head and alimentary canal. At 6 days post-microinjection, RT-qPCR assay showed that NcHig antibody treatment significantly increased the mean viral genome copies number in *N. cincticeps* heads (Figure 4A). However, NcHig antibody treatment did not affect viral load in the alimentary canal of *N. cincticeps* (Figure 4B). Immunofluorescence microscopy confirmed that RYSV propagation in the CNS was strongly increased after NcHig antibody treatment (Figure 4C). Together, these results confirm that the neuron-specific factor NcHig controls RYSV propagation in the CNS of *N. cincticeps*.

### The Antiviral Function of Hig Is Conserved in the Leafhopper *R. dorsalis*

**FIGURE 5:**
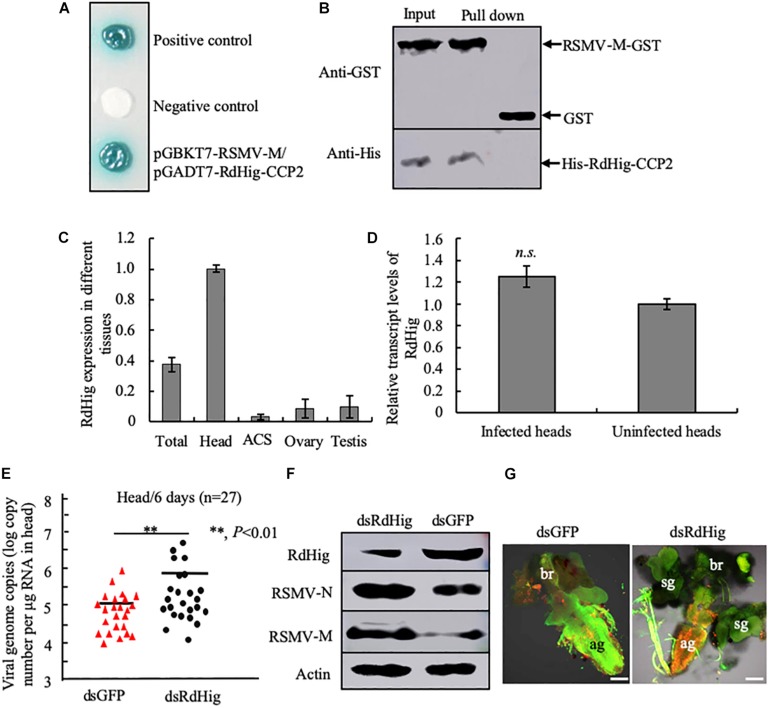
RdHig has antiviral activity during RSMV infection of the CNS of *R. dorsalis*. **(A)** Yeast two-hybrid assay to detect the interaction of RSMV M protein with the CCP2 domains of RdHig. **(B)** GST pull-down assay to verify the interaction of RdHig CCP2 domains with RSMV M protein. GST protein was used as the control. **(C)** RT-qPCR assay to detect RdHig transcript levels in different tissues of *R. dorsalis*. ACS, alimentary canal. Values are the means (±SE) of three biological replicates. **(D)** The transcript levels of RdHig expression in RSMV-infected or uninfected heads of *R. dorsalis*, as detected by an RT-qPCR assay. Values are the means (±SE) of three biological replicates. **(E)** RT-qPCR assay of viral genome copies of RSMV in the heads of *R. dorsalis* at 6 days post-microinjection of dsRNAs. RSMV genome copies in the individual heads (*n* = 27) of viruliferous *R. dorsalis* were calculated as the log of the copies number/μg RNA in head based on the standard curve for the RSMV N gene. *P*-values were estimated using a Student’s *t*-test. **(F)** The accumulation of RdHig, RSMV N, and RSMV M in the heads of viruliferous *R. dorsalis* at 6 days post-microinjection of dsRdHig and dsGFP, as detected by an immunoblot assay. Insect β-actin was used as the internal control. **(G)** RSMV infection of the CNS of viruliferous *R. dorsalis* at 6 days post dsRNA microinjection, as analyzed by an immunofluorescence assay. Virus-infected heads were immunolabeled with RSMV-M-rhodamine (red) and α-tubulin-FITC (green). ag, abdominal ganglion; br, brain; sg, salivary gland. Bars, 50 μm.

RSMV is a neurotropic rice cytorhabdovirus that invades the CNS of its leafhopper vector *R. dorsalis* (Yang et al., 2017a; Zhao et al., 2019). The CCP2 domains of a Hig homolog in *R. dorsalis* (RdHig) (GenBank accession no. MT043161) shared 92.27% similarity with NcHig CCP2 domains (Supplementary Figure S1B). Yeast two-hybrid assay indicated that RSMV M protein directly interacted with the CCP2 domains of a Hig homolog in *R. dorsalis* (RdHig) (Figure 5A). GST pull-down assay confirmed such interaction (Figure 5B). RT-qPCR assay also showed that RdHig is specifically expressed in the head of *R. dorsalis* (Figure 5C), and that its expression is not activated during RSMV infection (Figure 5D). Furthermore, RT-qPCR, immunoblots, and immunofluorescence microscopy all showed that the microinjection of dsRdHig significantly enhanced the propagation of RSMV in the CNS of *R. dorsalis* (Figures 5E–G). These results suggest that the Hig proteins have conserved antiviral roles in rice leafhoppers.

## Discussion

Many plant rhabdoviruses can invade, systemically infect, and propagate within the CNS of their insect vectors (Ammar and Hogenhout, 2008; Todd et al., 2010; Wang et al., 2019; Zhao et al., 2019). Leafhoppers are the natural vectors of RYSV and RSMV, and their brains are highly susceptible to rice rhabdovirus infection. However, such infections do not affect leafhopper cytopathology, implying that leafhopper brains have antiviral mechanisms that limit the viral burden to safe levels. Little is known about potential neuron-specific antiviral mechanisms in leafhoppers. In this study, we reveal a previous unreported antiviral mechanism that the leafhopper homologs of the neural factor Hig modulate the persistent infection of rice rhabdoviruses in the CNS of leafhopper vectors.

The CCP domains of Hig proteins are evolutionarily conserved, play important roles in neuron development, and interact with the surface of several pathogenic viruses (Casasnovas et al., 1999; Xiao et al., 2014, 2015; Nakayama et al., 2016). For example, the CCP domains of Hig homolog of *A. aegypti* interact with the surface of flaviviruses and effectively restrict flavivirus infection in the CNS of mosquitoes, preventing the lethal flaviviral infection of mosquitoes (Xiao et al., 2014, 2015). Here, we show that two rice rhabdoviruses RYSV and RSMV have also evolved a similar strategy by interaction of viral M proteins with the conserved CCP domains of Hig homologs of leafhoppers, efficiently controlling viral infection in the CNS of insect vectors. Many vector-borne viruses of agricultural or healthy importance such as reovirus, rhabdovirus, and flaviviruses are neurotropic and can persistently infect CNS of their insect vectors without causing significant cytopathology (Ammar and Hogenhout, 2008; Todd et al., 2010; Chen et al., 2011; Xiao et al., 2015; Wang et al., 2019; Zhao et al., 2019). We deduce that the involvement of Hig homologs in conferring resistance to the infection of vector-borne viruses in insect CNS may be a common phenomenon in nature.

The matrix molecules of distantly related rhabdoviruses M proteins have very similar coiled helical structures that condense the RNP core to assemble non-enveloped virions (Graham et al., 2008). We have shown that the expression of RYSV M protein in *Spodoptera frugiperda* (Sf9) cells resulted in the formation of such coiled helical tubule-like structures (Wang et al., 2019). Thus, it is possible that Hig CCP domains of *N. cincticeps* may directly interact with the rigid helical structures of RYSV M protein on the surface of non-enveloped virons in the CNS of insect vectors, but not with specific domains of RYSV M protein.

Previously, we have shown that the RYSV M protein directly interacted with α-tubulin, and thus, the non-enveloped RYSV particles can move along axon microtubules to facilitate long-distance viral spread in the CNS of *N. cincticeps* (Wang et al., 2019). Here, we further determine that the direct interaction of RYSV M protein with the CCP domains of Hig inhibits the extensive infection of the CNS by the virus, preventing the lethal viral infection of *N. cincticeps*. Taken together, it is possible that Hig may compete with α-tubulin for binding to the surface of non-enveloped RYSV particles, thereby preventing the excessive spread of RYSV in CNS and limiting viral burden to a safe level in the CNS. Thus, the M protein-binding ability of Hig may be essential for its antiviral activity and for its ability to modulate the persistent infection of RYSV in the CNS of *N. cincticep*. However, RYSV infection of the leafhopper CNS does not change Hig expression level, suggesting that the ability for Hig to bind and restrict the spread of non-enveloped RYSV particles is not strongly activated during viral persistent infection of insect CNS. Interestingly, the neuron-specific factor Hig homolog of leafhopper *R. dorsalis* also demonstrates a prominent antiviral role during the persistent infection of the RSMV in the CNS. Our results reveal an evolutionarily conserved antiviral mechanism for Hig to mediate the persistent infection of rice rhabdoviruses in the CNS of leafhopper vectors, and thus may facilitate rhabdovirus transmission in nature.

Interestingly, the antiviral activity of Hig is limited to the CNS and has no effect in the intestines of leafhopper vectors. Usually, the propagation of plant viruses in the intestines of insect vectors triggers a conserved small interfering RNA (siRNA) antiviral pathway. For example, a virus-induced siRNA antiviral pathway effectively restricts the replication of southern rice black streaked dwarf virus, a plant reovirus, in the midgut epithelium of the small brown planthopper, which ultimately affects the capacity for viral transmission (Lan et al., 2015). Tomato yellow leaf curl virus, a begomovirus, can activate autophagy in the midgut of whitefly vectors to promote resistance to viral infection (Wang et al., 2016). By contrast, plant viruses may also manipulate vector immune systems for their efficient infection in the midgut. For example, autophagy and apoptosis can be activated by rice gall dwarf virus, a plant reovirus, to promote viral replication in the midgut of leafhopper vectors (Chen et al., 2017; Chen Q. et al., 2019). The nucleoprotein of rice stripe virus, a tenuivirus, promotes its replication by activating the c-Jun N-terminal kinase pathway in the midgut of its planthopper vector (Wang et al., 2017). The best studied insect innate immune system pathways at least include Toll, Imd, JAK-STAT, Nf-kB, RNAi, apoptosis, and autophagy (Wei et al., 2018). How these insect innate immune responses cooperate with Hig-mediated antiviral response to modulate the persistent viral infection in vector CNS would be investigated in the future. Exploring the role of insect antiviral factors in CNS infections will broaden our understanding of the sophisticated interactions between plant viruses and their vectors and may provide insights into novel approaches to attenuate viral epidemics.

## Data Availability Statement

The raw data supporting the conclusions of this manuscript will be made available by the authors, without undue reservation, to any qualified researcher.

## Ethics Statement

The mouse polyclonal antibody against the NcHig was prepared by the Beijing Protein Innovation Company, which was approved by the Beijing Municipal Science and Technology Commission.

## Author Contributions

HW and TW conceived and designed the experiments. HW, YL, LM, ZW, CH, and PZ performed the experiments. HW, HC, DJ, QC, XZ, and TW analyzed the data. HW and TW wrote the manuscript. All authors read and approved the final manuscript.

## Conflict of Interest

The authors declare that the research was conducted in the absence of any commercial or financial relationships that could be construed as a potential conflict of interest.

## Footnotes

1 http://smart.embl-heidelberg.de/smart/set_mode.cgi?GENOMIC=1

2 http://pfam.xfam.org/

## Supplementary Material

FIGURE S1 **(A)** Percentages of amino acid identities between NcHig and *A. aegypti* Hig (AaHig) or *D. melanogaster* Hig (DmHig). **(B)** Unrooted phylogenetic tree of insect Hig proteins constructed using the neighbour-joining (NJ) method based on the alignment of insect Hig protein sequences downloaded from NCBI database. The bootstrap values of 5000 replicates are indicated at the branch nodes. Bar, 0.1 represents 10 differences per 100 amino acids.

FIGURE S2 Preparation of NcHig mouse polyclonal antibody. **(A,B)** The prokaryotic expression of the domains of NcHig (IG domain-CCP2 repeats, 293–631 aa). The prokaryotic expression proteins were detected by Coomassie brilliant blue staining with adding IPTG or without adding IPTG in the induction progress **(A)** and immunoblot **(B)**. **(C)** Total proteins from the heads of *N. cincticeps* and *R. dorsalis*, detected separately using NcHig-specific antibody.

FIGURE S3 Survival rates of dsGFP- or dsNcHig-treated non-viruliferous or viruliferous *N. cincticeps.* The microinjected *N. cincticeps* individuals (*n* = 70) were maintained with rearing and daily observed for calculation of survival rates. The data were statistically analyzed from three biological repeats. *P*-values were estimated using a Student’s *t*-test.

TABLE S1 The primers used in the study.

TABLE S2 Candidate proteins of *N. cincticeps* interacting with RYSV M protein screened with yeast two-hybrid system.
