# Supplementary figures and images for: A Neuron-Specific Antiviral Mechanism Modulates the Persistent Infection of Rice Rhabdoviruses in Leafhopper Vectors
Source: Front Microbiol. 2020 Apr 17;11:513. doi: 10.3389/fmicb.2020.00513 (PMC7180231; doi:10.3389/fmicb.2020.00513)

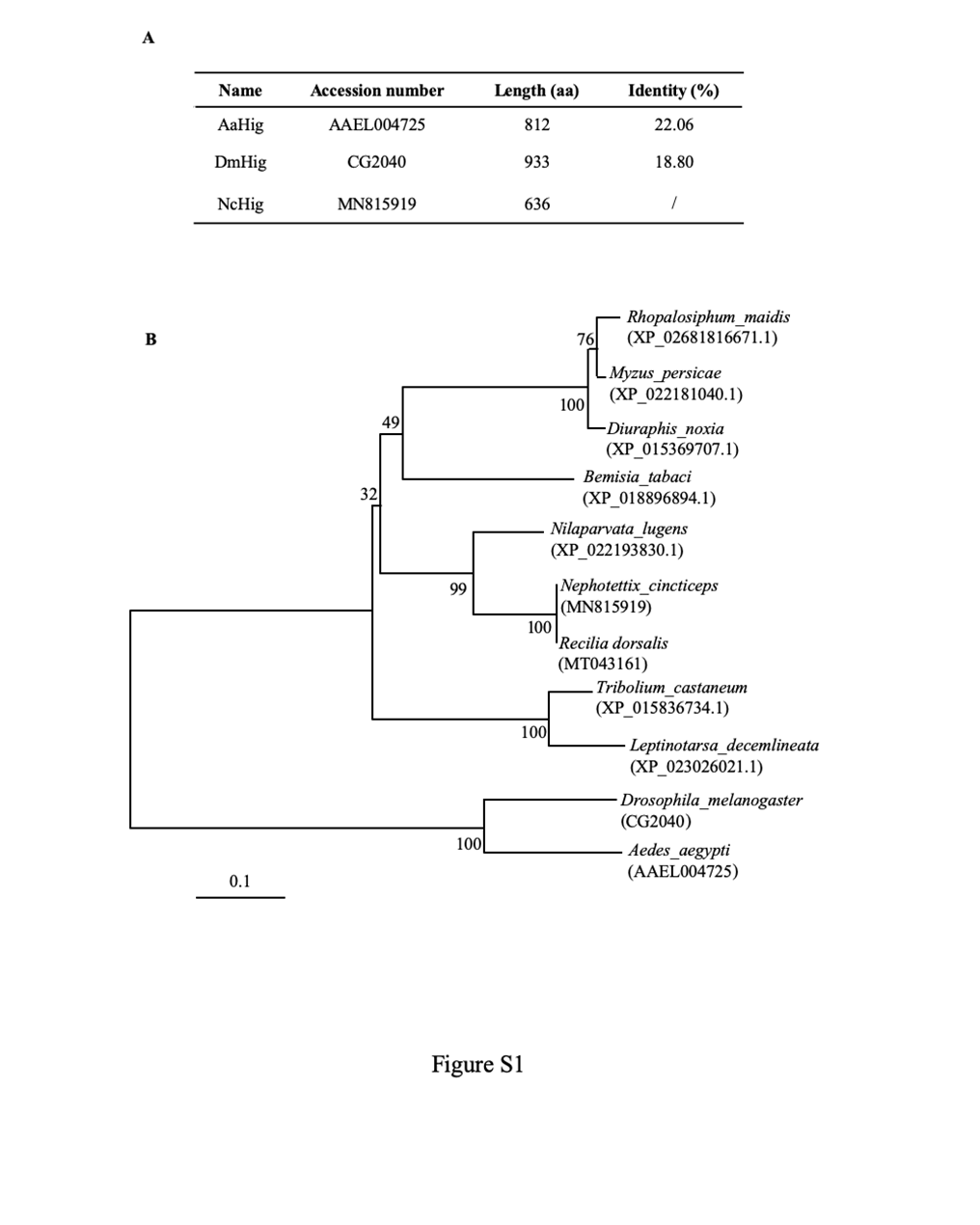

Supplement: FIGURE S1 — (A) Percentages of amino acid identities between NcHig and A. aegypti Hig (AaHig) or D. melanogaster Hig (DmHig). (B) Unrooted phylogenetic tree of insect Hig proteins constructed using the neighbour-joining (NJ) method based on the alignment of insect Hig protein sequences downloaded from NCBI database. The bootstrap values of 5000 replicates are indicated at the branch nodes. Bar, 0.1 represents 10 differences per 100 amino acids. [file Image_1.TIFF]

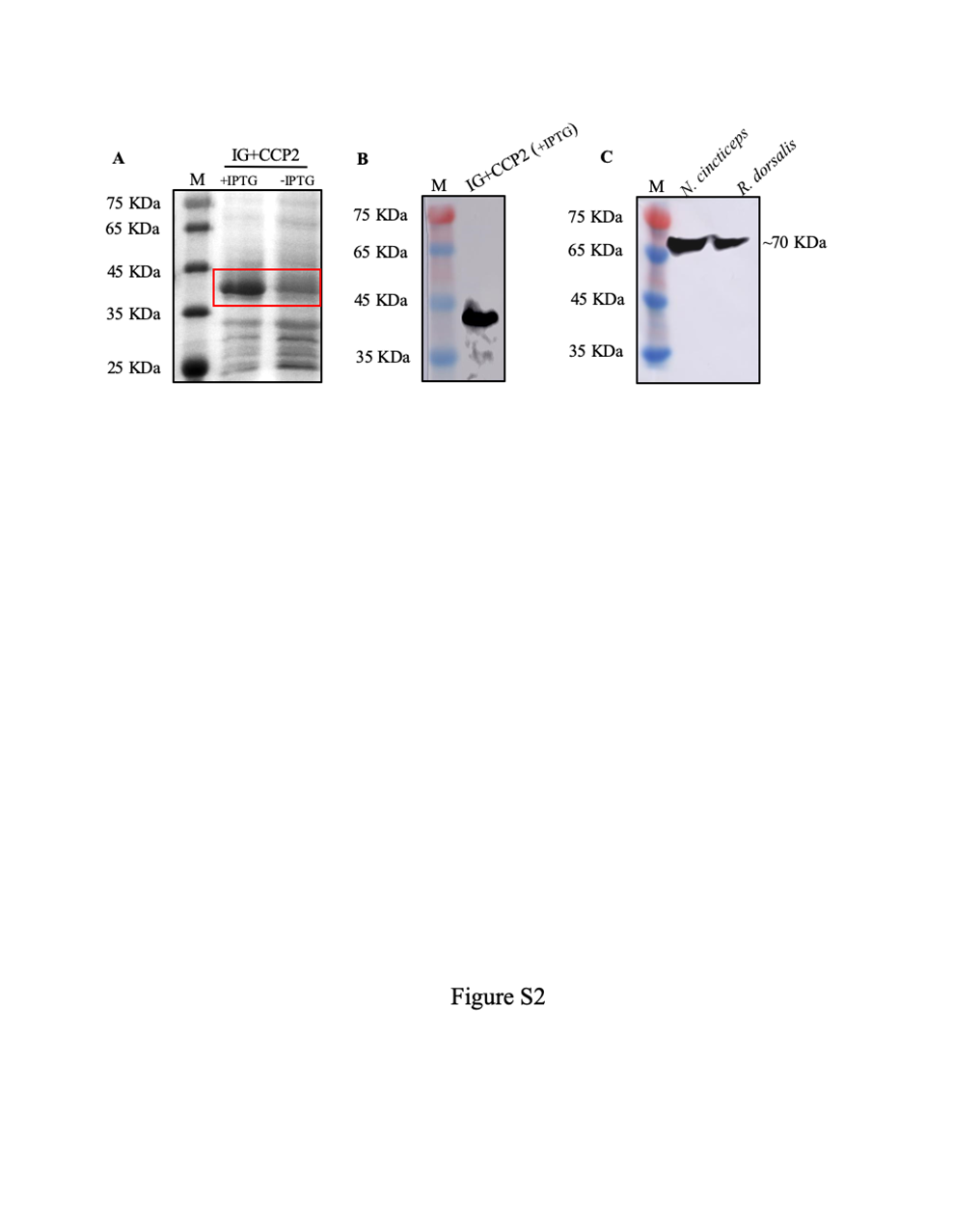

Supplement: FIGURE S2 — Preparation of NcHig mouse polyclonal antibody. (A,B) The prokaryotic expression of the domains of NcHig (IG domain-CCP2 repeats, 293–631 aa). The prokaryotic expression proteins were detected by Coomassie brilliant blue staining with adding IPTG or without adding IPTG in the induction progress (A) and immunoblot (B). (C) Total proteins from the heads of N. cincticeps and R. dorsalis, detected separately using NcHig-specific antibody. [file Image_2.TIFF]

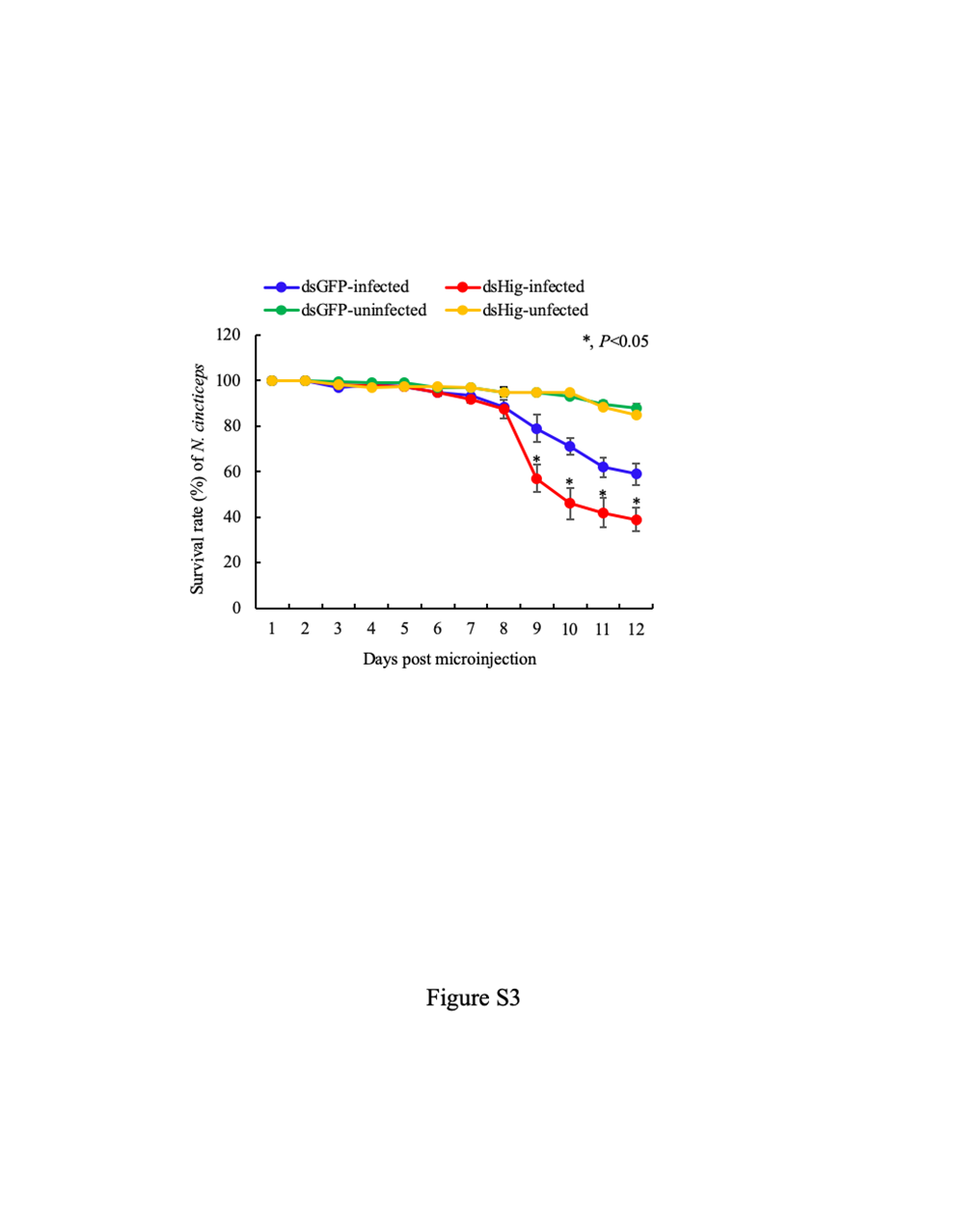

Supplement: FIGURE S3 — Survival rates of dsGFP- or dsNcHig-treated non-viruliferous or viruliferous N. cincticeps. The microinjected N. cincticeps individuals (n = 70) were maintained with rearing and daily observed for calculation of survival rates. The data were statistically analyzed from three biological repeats. P-values were estimated using a Student’s t-test. [file Image_3.TIFF]
