# Supplementary material for: A Neuron-Specific Antiviral Mechanism Modulates the Persistent Infection of Rice Rhabdoviruses in Leafhopper Vectors
Source: Front Microbiol. 2020 Apr 17;11:513. doi: 10.3389/fmicb.2020.00513 (PMC7180231; doi:10.3389/fmicb.2020.00513)
Supplement: TABLE S1 — The primers used in the study. [file Table_1.DOCX]

Table S1 The primers used in the study

| **Name** | **Primer sequence** |
| --- | --- |
| Actin-F-qPCR | GGGATACAGTTTCACCACG |
| Actin-R-qPCR | GACACCTGAATCGCTCGT |
| EF1-F-qPCR | CAGTGAGAGCCGTTTTGAG |
| EF1-R-qPCR | AGGGCATCTTGTCAGAGGGC |
| NcHig-F-qPCR | AAGGAGAATGGGATGGGCAA |
| NcHig-R-qPCR | AGTACCGAACCTCCGAATCC |
| RdHig-F-qPCR | TCAAGAGAATGGCAGGTGGT |
| RdHig-R-qPCR | CTGCGTTTGACCACTTTCCA |
| RYSV-N-F-qPCR | AGTATGCCCAACTTGCCAGG |
| RYSV-N-R-qPCR | CATTCGTTCAACCGGCATCC |
| RSMV-N-F-qPCR | CAGACCTGAACTGTCCCGAG |
| RSMV-N-R-qPCR | GACGAATACTCACCTGCGGA |
| dsNcHig-F | T7-CTCCACTTGCTTATTCCGTA |
| dsNcHig-R | T7-TGTTCATCTTGGTGTTCTGT |
| dsGFP-F | T7-CTTGTTGAATTAGATGGTGATGTT |
| dsGFP-R | T7-TTTCGAAAGGGCAGATTGT |
