# Supplementary material for: A Neuron-Specific Antiviral Mechanism Modulates the Persistent Infection of Rice Rhabdoviruses in Leafhopper Vectors
Source: Front Microbiol. 2020 Apr 17;11:513. doi: 10.3389/fmicb.2020.00513 (PMC7180231; doi:10.3389/fmicb.2020.00513)
Supplement: TABLE S2 — Candidate proteins of N. cincticeps interacting with RYSV M protein screened with yeast two-hybrid system. [file Table_2.DOCX]

**Table S2 Candidate proteins of *N. cincticeps* interacting with RYSV M protein screened with yeast two-hybrid system**

| NO. | Accession number | BLASTX results | species | |
| --- | --- | --- | --- | --- |
| 1 | XP_015836735 | Complement factor H isoform X4 | | *Tribolium castaneum* |
| 2 | XP_012060436.1 | Iron/zinc purple acid phosphatase-like protein | | *Apis mellifera* |
| 3 | XP_001147491.1 | Immunoglobulin superfamily member 3 isoform X1 | | *Pan troglodytes* |
| 4 | XP_003817895.1 | GTP-binding nuclear protein Ran | | *Pan paniscus* |
| 5 | XP_008547621.1 | Alpha-tocopherol transfer protein-like | | *Microplitis demolitor* |
| 6 | AGZ15210.1 | Phosphoesterase 62, partial | | *Laodelphax striatella* |
| 7 | XP_970818.1 | Bifunctional arginine demethylase and lysyl-hydroxylase JMJD6 | | *Tribolium castaneum* |
| 8 | EEB20328.1 | Aarginine/serine-rich splicing factor | | *Pediculus humanus corporis* |
| 9 | XP_012256060.1 | EH domain-containing protein 3 | | *Athalia rosae* |
| 10 | AIV98130.1 | tubulin alpha-2 | | *Laodelphax striatella* |
| 11 | XP_012267441.1 | PDZ and LIM domain protein Zasp isoform X2 | | *Athalia rosae* |
| 12 | KDR11195.1 | Rap1 GTPase-GDP dissociation stimulator 1-B | | *Acromyrmex echinatior* |
| 13 | EFA00103.1 | Similar to Rab-protein 10 CG17060-PA | | *Tribolium castaneum* |
| 14 | BAN20569.1 | Interferon-related developmental regulator 1-like | | *Bombus terrestris* |
| 15 | XP_003244558.1 | Peroxisomal multifunctional enzyme type 2 isoform X1 | | *Acyrthosiphon pisum* |
| 16 | XP_966692.1 | Transitional endoplasmic reticulum ATPase TER94 | | *Tribolium castaneum* |
| 17 | XP_008190203.1 | Tubulin beta chain-like | | *Laodelphax striatella* |
| 18 | KDR10939.1 | Cytochrome P450 9e2 | | *Zootermopsis nevadensis* |
| 19 | KDR22965.1 | Protein kinase C and casein kinase substrate in neurons protein 2 | | *Zootermopsis nevadensis* |
| 20 | XP_001845679.1 | Dihydrolipoamide succinyltransferase component of 2-oxoglutarate dehydrogenase | | *Culex quinquefasciatus* |
| 21 | BAN20148.1 | Leucyl aminopeptidase | | *Riptortus pedestris* |
| 22 | AHB33468.1 | T-complex protein subunit alpha | | *Locusta migratoria* |
| 23 | XP_011344499.1 | PTB domain-containing engulfment adapter protein | | *Megachile rotundata* |
| 24 | XP_001604718.2 | Ras-related protein Rab6 isoform X2 | | *Nasonia vitripennis* |
| 25 | EDS39731.1 | 78 kDa glucose-regulated protein | | *Culex quinquefasciatus* |
| 26 | ACU25861.1 | Sarco/endoplasmic reticulum calcium ATPase | | *Bombyx mori* |
| 27 | XP_012280872.1 | RNA-binding protein 45 | | *Orussus abietinus* |
